# Case Report: Activated phosphoinositide 3-kinase δ syndrome mimicking Hyper-IgM syndrome: early hepatosplenomegaly as a key diagnostic clue

**DOI:** 10.3389/fimmu.2026.1881308

**Published:** 2026-08-28

**Authors:** Anh T. V. Nguyen, Giang D. Nguyen, Quynh T. T. Bui, Mai T. P. Nguyen, Anh P. Ha, Hanh H. Chu, Ha T. Dang, Cuc P. Hoàng, Van D. Nguyen, Tung V. Cao, Dien M. Tran

**Affiliations:** 1 Vietnam National Children’s Hospital, Vietnam National Hospital of Pediatrics, Hanoi, Vietnam; 2 Department of Hematology and Oncology, Children's Hospital 2, Ho Chi Minh City, Vietnam

**Keywords:** activated phosphoinositide 3-kinase δ syndrome, APDS, hepatosplenomegaly, lymphoproliferation, sirolimus

## Abstract

**Background:**

APDS is a combined immunodeficiency disorder, characterized by impaired antibody production and lymphoproliferation, with a high risk of malignancy and autoimmunity. The disease may be caused by autosomal dominant gain-of-function variants in the *PIK3CD* or loss-of-function variants in the *PIK3R1* gene.

**Case summary:**

We report an 11-year-old girl with a history of recurrent respiratory infections, lymphoproliferation, and hepatosplenomegaly from early childhood. She was diagnosed with Hyper-IgM syndrome (HIGM) because of decreased IgA and IgG but increased IgM, and was treated with immunoglobulin replacement therapy, but there was no improvement in respiratory symptoms, and hepatosplenomegaly remained marked. Immunologic testing showed that, in addition to a reduced peripheral B-cell count, there was also a reduced CD4^+^ T-cell count and an inverted CD4/CD8 ratio, suggesting a diagnosis other than classical HIGM. The patient was diagnosed with APDS, confirmed by whole-exome sequencing, identifying the *PIK3CD* c.3061G>A p.(Glu1021Lys) variant.

**Outcome:**

The patient maintained on immunoglobulin replacement therapy and corticosteroid therapy, with subsequent addition of sirolimus, and showed a rapid response with improvement in chronic productive cough, enlarged lymph nodes, and hepatosplenomegaly after 2 months. After 6 months of treatment, lymphodenopathy had resolved, and the liver and spleen returned to normal size.

**Conclusion:**

Early hepatosplenomegaly and lymphadenopathy are important warning signs of APDS in children with a Hyper IgM-like phenotype. This is particularly relevant in patients with CD4^+^ T-cell lymphopenia, an inverted CD4/CD8 ratio, and marked B-cell lymphopenia. Sirolimus improved lymphoproliferative manifestations and partially controlled chronic respiratory and gastrointestinal disease but did not eliminate the need for immunoglobulin replacement therapy.

## 1 Introduction

Inborn errors of immunity are a group of rare diseases caused by genetic abnormalities. According to the 2024 IUIS classification, there are more than 550 phenotypes caused by over 500 genes (1). This group of diseases exhibits a broad spectrum of clinical manifestations, not limited to recurrent severe infections due to immunodeficiency but also including features of immune dysregulation such as autoimmunity, autoinflammation, allergy, and malignancy.

APDS is one of the syndromes causing severe humoral immunodeficiency combined with immune dysregulation. The disease is caused by heterozygous gain-of-function variants in the *PIK3CD* or dominant loss-of-function variants in the *PIK3R1*. Both genes encode components of PI3Kδ (2, 3).

PI3Kδ is predominantly expressed in lymphocytes and plays a central role in T- and B-cell signaling. Gain-of-function variants in *PIK3CD* result in constitutive activation of the PI3Kδ–AKT–mTOR pathway, even in the absence of appropriate upstream stimulation. This dysregulated signaling impairs lymphocyte differentiation, promotes T-cell senescence and B-cell maturation defects, and ultimately leads to chronic lymphoproliferation, reduced B-cell count, and defective antibody production (2).

APDS due to gain-of-function variants in the *PIK3CD* gene causes diverse clinical manifestations, including immunodeficiency combined with immune dysregulation, autoimmune manifestations, autoinflammation, and lymphoproliferation. Besides severe and recurrent infections, especially in the respiratory tract with a high rate of bronchiectasis, patients have manifestations of chronic benign lymphoproliferation in many different organs, including: lymphadenopathy, hepatosplenomegaly, and possibly lymphoma (3, 4). Autoimmune manifestations such as autoimmune cytopenias (immune thrombocytopenia, autoimmune hemolytic anemia, autoimmune neutropenia) and autoinflammatory manifestations and gastrointestinal involvement are common: prolonged diarrhea, malabsorption, enteropathy reflecting the state of immune dysregulation in the gut. In addition, herpesvirus infections, particularly CMV and EBV, are common in patients with APDS and are clinically significant because they may lead to chronic lymphoproliferation and EBV-driven lymphoid disease, thereby increasing the risk of lymphoma (4, 5).

Treatment of APDS usually includes immunoglobulin replacement therapy to avoid infections, as well as immunosuppressive agents to regulate autoimmunity and lymphoproliferation such as sirolimus (rapamycin), tacrolimus, rituximab. The study by Maccari and colleagues based on a multicenter analysis from ESID-APDS Registry data showed that sirolimus brought moderate to good clinical benefit in most patients. The mechanism is explained by sirolimus binding to FKBP12 to form the sirolimus-FKBP12 complex; this complex binds to mTORC1, thereby inhibiting kinase activity and blocking activation of effector T-cell lymphoproliferation. The study showed that the main effect was a reduction in lymphoproliferation. Effects on inflammatory bowel disease and autoimmune cytopenias remain limited. The study also recorded that early-onset bronchiectasis did not reverse lung structural damage after treatment (6).

In 2023, Rao and colleagues published the results of a phase 3 randomized, placebo-controlled trial of the selective PI3Kδ inhibitor leniolisib, showing significant immunologic improvement in patients with APDS. The drug is a PI3Kδ-targeted therapy, reducing the overactivated PI3Kδ signaling due to *PIK3CD or PIK3R1* variants. The trial demonstrated long-term effects, not only controlling lymphoproliferation and immune dysregulation (reducing T-cell senescence), restoring the balance of B-lymphocyte populations but also reducing dependence on immunoglobulin replacement therapy (7).

Hematopoietic stem cell transplantation (HSCT) is a potentially curative treatment option for patients with severe or progressive APDS, particularly those with lymphoma or refractory disease, but associated with substantial morbidity and mortality (8, 9).

Aim of the article: To highlight the critical importance of differentiating HIGM from activated phosphoinositide 3-kinase δ syndrome, in order to avoid diagnostic misclassification and enable timely implementation of appropriate targeted therapy.

## 2 Case presentation

An 11-year-old girl was first referred to our hospital at 9 years of age. Her family history was unremarkable, with one healthy 3-year-old younger brother. She had been diagnosed with HIGM at 6 years of age. At 2 months of age, her An 11-year-old girl was first referred to our hospital at 9 years of age. At 2 months of age, her mother noticed a small lymph node in the left cervical region. Several days later, the infant developed irritability and was evaluated at a local hospital. The cervical lymph node was considered reactive to BCG vaccination, and no further investigations, including immunological evaluation, were performed. There was no family history suggestive of primary immunodeficiency or lymphoproliferative disease. At 13 months of age, she had her first episode of pneumonia, followed by recurrent infections, including recurrent pneumonia (3 episodes per year), lung abscess, otitis media, multiple episodes of infectious diarrhea and one episode of parotitis. From 4 years of age, she developed cervical lymphadenopathy, hepatomegaly, and anemia but remained untreated. At 6 years of age, she developed diarrhea, progressive hepatosplenomegaly, lymphadenopathy, worsening anemia. She was evaluated at a local hospital and diagnosed with bronchiectasis and HIGM. She was treated with regular IVIG 400–500 mg/kg every 4–5 weeks for more than 2 years with IgG trough level maintained at 4–5 g/L. However, bronchiectasis did not improve, and she continued to have daily productive cough. Serial complete blood counts obtained during the year before referral consistently demonstrated normal absolute lymphocyte counts (1.9–2.7 × 10^9^ cells/L) without persistent lymphocytosis. She was transferred to our hospital at 9 years of age because of high fever, prolonged watery diarrhea, pneumonia, multiple cervical and axillary lymph nodes measuring 1–2 cm, and marked hepatosplenomegaly (liver and spleen were palpable 5 cm below the costal margin), and tense abdominal distension. The patient was malnourished, with weight-for-age z score -3.5 SD, height-for-age z score -1.5 SD, and had bilateral leg edema.

On admission, the complete blood count showed normocytic normochromic anemia with a hemoglobin level of 7.0 g/dL and mild age-adjusted lymphopenia (1.79 x 10^9^ cells/L). Serum immunoglobulin levels showed decreased immunoglobulin levels, including IgA 0.06 g/L, IgG 0.5 g/L, IgM 12.02 g/L, and IgE was 2.1 IU/mL. Lymphocyte subset analysis showed a normal absolute CD3+ T-cell count of 1763/μL (85%), but markedly reduced CD4^+^ T -cell count of 282/μL (13.60%), while the CD8^+^ T-cell count was normal at 1342/μL (64.59%), with an inverted CD4/CD8 ratio. The B-cell count was reduced, with CD19^+^ cells: 35/μL (1.66%), while CD56^+^NK cells were 279/μL (13.42%). Thus, the notable difference from HIGM was that the patient had low CD4^+^ T cells, an inverted CD4/CD8 ratio, and reduced B cells. The patient's hematologic and immunologic investigations at different time points are summarized in **Table 1**.

**Table 1:** Immunological investigations.

Parameter	Six years old	Nine years old *(on admission) *	Before sirolimus	Ten years old (1 year after sirolimus)	11 years old (2 years after sirolimus)
WBCs (10^9^ cells/L)	6.6	6.7	8.22	7.51	8.29
Hemoglobin (g/dL)	7.5	7.0	7.2	8.4	9.8
Neutrophils (10^9^ cells/L)	1.6	4.1	5.57	4	4.53
Lymphocytes (10^9^ cells/L)	4.3	2.01	2.00	2.57	2.82
Platelets (10^9^ cells/L)	342	354	519	414	393
Lymphocyte subsets (normal range, unit)					
CD3^+^ T cells (1200 – 2600,/uL)	1718	1763	1685	2307	2501
CD3^+^%	77.90	84.83	84.92	81.60	83.69
CD3^+^CD4^+^ T cells (650-1500,/µL)	*223*	*282*	*308*	*439*	*404*
CD3^+^CD4^+^%	10.10	13.60	15.53	15.54	13.51
CD3^+^CD8^+^ T cells (370-1100, µL)	1265	1342	1283	1685	1858
CD3^+^CD8^+^%	57.3	64.59	64.67	58.64	62.19
*CD4^+^/CD8^+^ (1.5)*	*0.17*	*0.21*	*0.24*	*0.26*	*0.21*
CD19^+^ B cells (270-860,/µL)	222	34.53	88.41	235	201
CD19^+^%	10.40	1.66	4.46	8.31	6.73
CD56^+^ NK cells (100-480,/µL)	230	279	210.76	282	280
CD56%	10.60	13.42	10.62	9.96	9.37
Immunoglobulins (normal range)					
IgM (0.5 - 1.6 g/L)	6.42	12.02	13.41	15.22	12.04
IgG (5.5 – 11.5 g/L)	*0.01*	*0.50*	*4.18*	*5.30*	*5.72*
IgA (0.4 – 1.6 g/L)	0.49	0.06	0.04	0.13	0.10

**Figure 1:**
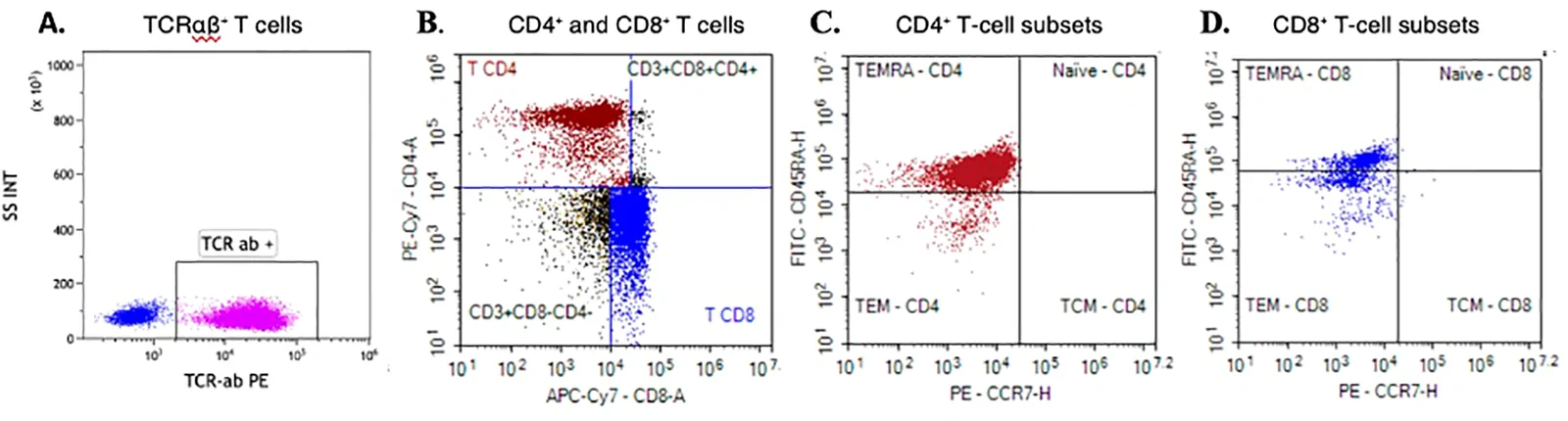
TCRαβ+ double-negative T cells and T-cell differentiation profile after 2 years of sirolimus treatment. T cell subsets based on CD45RA and CCR7 expression: Naïve (CD45RA^+^CCR7^+^), Central memory/TCM (CD45RA^−^CCR7^+^), Effector memory/TEM (CD45RA^−^CCR7^−^), Terminally differentiated/TEMRA (CD45RA^+^CCR7^−^). Decreased Naïve and increased TEMRA.

At two years follow-up, flow cytometric analysis showed that TCRαβ^+^ double-negative T cells accounted for 0.9% of total lymphocytes and 1.09% of CD3^+^ T cells. Despite marked clinical improvement, the proportion of CD4^+^ T lymphocytes remained low at 13.5%. Subset analysis showed naive CD4^+^ T cells of 0 and naive CD8^+^ T cells of 2 cells/µL (0% and 0.16%), with a marked expansion of TEMRA CD4^+^ (91.85%) and TEMRA CD8^+^ (41.77%) populations. The immunophenotypic findings are summarized in **Figure 1**.

Abdominal CT revealed hepatosplenomegaly with homogeneous hepatic and splenic parenchyma. A large lobulated hypodense lesion (59 × 55 mm) was indentified adjacebt to the head the pancreas head region, and multiple enlarged abdominal lymph nodes.

**Figure 2:**
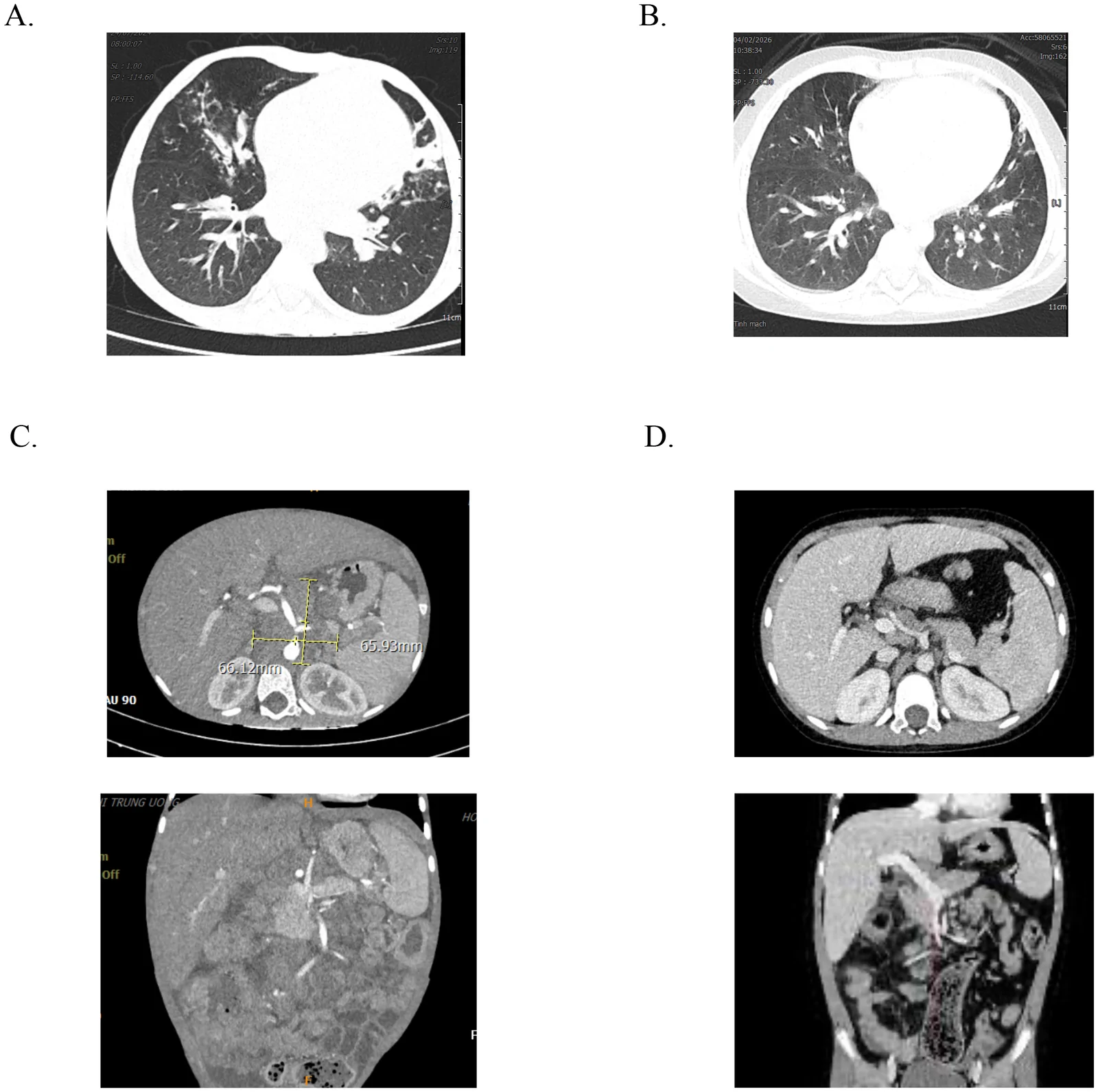
Radiological findings pre-treatment and after 2 years of sirolimus treatment. **(A)** Pre-treatment chest CT: Bronchiectasis with bilateral involvement and mediastinal and axillary lymphadenopathy (up to 19 × 31 mm and 32 × 21 mm). **(B)** Chest CT after 2 years of sirolimus: Mild improvement of bronchiectasis with reduced lymphadenopathy; residual paratracheal nodes (11 × 17 mm). **(C)** Baseline abdominal CT: Hepatosplenomegaly with abdominal lymphadenopathy (up to 36 × 20 mm) and a lobulated hypodense lesion adjacent to the pancreatic head (59 × 55 mm). **(D)** Abdominal CT after 2 years of sirolimus: Resolution of hepatosplenomegaly with residual calcified adjacent to the pancreatic head (6 × 7 mm).

The lesion was located adjacent to the pancreatic head and was initially suspected to be a pancreatic-head mass. However, pancreatic enzyme levels remained normal on two separate measurements. Multidisciplinary review of the imaging findings suggested that the lesion represented confluent lymphoid tissue adjacent to the pancreatic head rather than a pancreatic parenchymal lesion. Representative imaging findings are shown in **Figure 2**.

A laparoscopic biopsy was performed because of suspected lymphoma. However, given the clinical characteristics of early-onset lymphadenopathy from 2 months of age, persistent lymphadenopathy accompanied by hepatosplenomegaly from 4 years of age, and the absence of features suggestive of malignancy, such as changes in size, general condition, or pain, lymphoma was considered unlikely. The biopsy was reviewed in consultation with pathologists at the National Institutes of Health (USA), and showed benign follicular hyperplasia with preserved nodal architecture, no evidence of malignancy. Immunohistochemical staining showed a balanced T-cell population, with CD4/CD8, and no evidence of T-cell monoclonality. PD-1 positivity in the germinal center demonstrated the presence of physiological T follicular helper cells. CD20-positive B cells were distributed appropriately in the germinal center, and CD21 positivity confirmed that the follicular dendritic cell network remained intact. BCL-2 and BCL-6 were negative, helping to exclude follicular lymphoma. κ and λ light chains showed polytypic expression, supporting a reactive process and arguing against lymphoma. EBV PCR in peripheral blood was positive at 300 copies/mL. Taking all these data together, there was no evidence supporting the diagnosis of lymphoma, and lymphoma was excluded at the time of biopsy. Representative histopathological images were not available for publication. Infectious diseases were excluded: tuberculosis (by Gene-Xpert, AFB, MODS for tuberculosis in sputum and gastric aspiration. Testing for CMV, HHV-6, hepatitis A, B, and C viruses was negative. At the time of diagnosis, EBV PCR was weakly positive (300 copies/mL). Quantitative EBV PCR was monitored every 6 months after treatment initiation and remained negative throughout the follow-up period.

The patient underwent whole-exome sequencing (WES), detecting a c.3061G>A (p.Glu1021Lys) variant in the *PIK3CD* gene. This variant was classified as likely pathogenic according to ACMG/AMP variant classification guidelines (PM6, PM2, PM1, PP2, PP5). Structural analysis suggested that this variant position lies in a highly conserved catalytic domain across species. In addition, the change from the amino acid glutamic acid (negatively charged) to the amino acid lysine (positively charged) altered protein function. This variant has been proven to be a dominantly inherited variant and to increase protein function (gain-of-function - GOF). Parental Sanger sequencing did not detect the variant, suggesting a *de novo* occurrence.

Immunoglobulin replacement therapy was initiated with IVIG at 1 g/kg at the first admission due to severe hypogammaglobulinemia (IgG 0.5 g/L). This was followed by maintenance therapy with subcutaneous immunoglobulin (ScIG) at a dose of 600 mg/kg/month. The patient responded to prednisolone, started at 1 mg/kg/day. Diarrhea resolved after one week. Prednisolone was continued at this dose for 4 weeks, then gradually tapered to 0.75 mg/kg/day. Prophylactic azithromycin and fluconazole were also initiated.

At 1.5 months after treatment initiation, splenomegaly had decreased, and lymph nodes had reduced to small (approximately 0.5 cm) nodes in the cervical and inguinal regions. The patient gained 3 kg. However, hepatomegaly worsened, with abdominal distension. Ultrasound showed an enlarged liver measuring 158 mm. Sirolimus was initiated at 3 mg/m^2^ on day 1, followed by 1–2 mg/m^2^/day, targeting a trough level of 6–8 ng/mL, and prednisolone was gradually tapered. After 2 months of sirolimus therapy, the liver was palpable 3 cm below the costal margin, and the spleen was just palpable at the costal margin. After 6 months of treatment, both liver and spleen returned to normal size.

**Figure 3:**
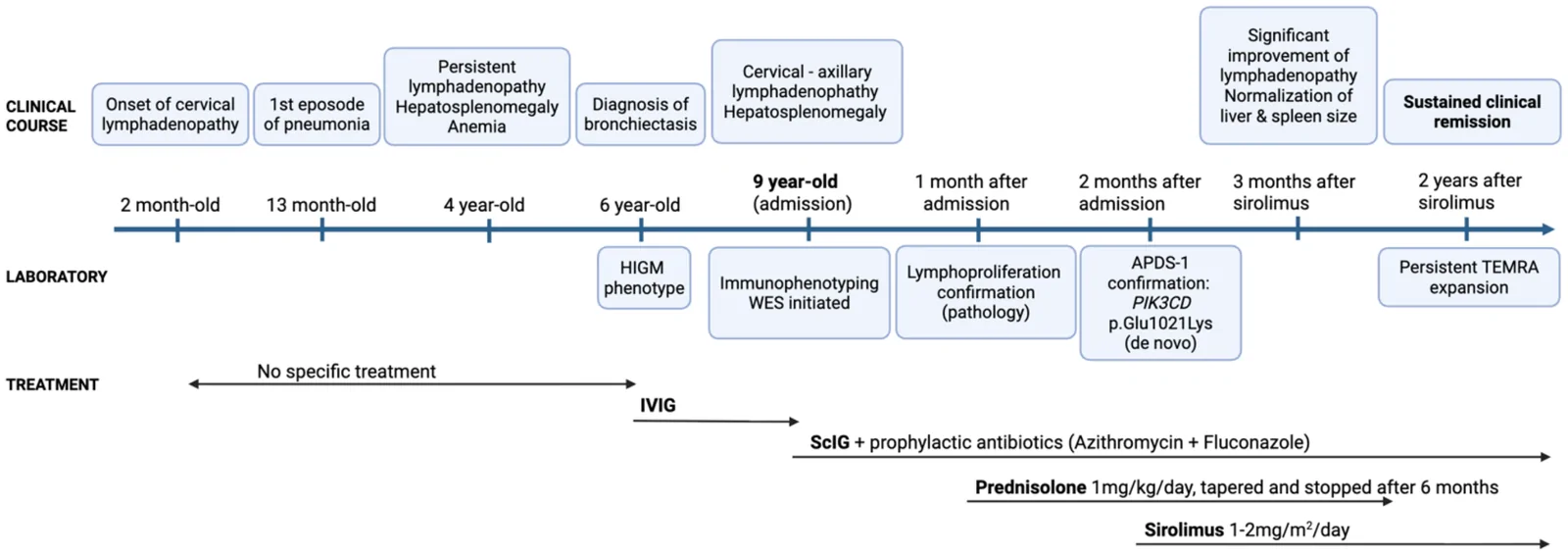
Timeline of the patient’s clinical course.

Respiratory symptoms also improved significantly. The patient no longer had productive cough, and chest CT showed improvement, with decreased bronchial wall thickening and less extensive bronchiectatic change. The patient's clinical course from infancy to diagnosis and follow-up is summarized in **Figure 3**.

## 3 Discussion

We report a case of APDS with the initial diagnosis of HIGM, with clinical manifestations of early-onset recurrent respiratory infections, elevated IgM with low IgA and IgG as classic manifestations of HIGM (3, 10). However, this patient had many clinical features atypical for HIGM and the initial diagnosis needed reconsideration. Notably, the patient had lymphadenopathy from 2 months of age, then hepatosplenomegaly and lymphadenopathy from 4 years of age. The patient exhibited lymphoproliferation inconsistent with HIGM, and the lymphadenopathy in our patient appeared as early as 2 months of age, earlier than most APDS reports in the literature.

The report by Basso and colleagues, in the journal *Blood*, demonstrated that CD40 signaling is an important factor for B-cell differentiation and germinal center formation. Therefore, HIGM patients exhibit disruption of this pathway, so germinal centers are usually poorly developed. Consequently, lymphoid tissue is usually small or normal, without manifestations of chronic lymphoproliferation (10–12). Therefore, early and prolonged lymphadenopathy in our patient is a sign not consistent with classical HIGM.

Besides that, in the evaluation of cellular immunity, our patient showed clear and persistent reduction of CD4^+^ T-cell count, inversion of the CD4^+^/CD8^+^ ratio, along with a reduced peripheral B-cell count, consistent with the mechanism of premature T-cell senescence and B-cell differentiation disorder due to excessive activation of the PI3Kδ–AKT–mTOR axis as described by Lucas (2). This represents a key distinguishing feature between HIGM and APDS. The presence of very early, large hyperplastic lymph nodes, together with chronic and marked hepatosplenomegaly, supports an alternative diagnosis to HIGM and suggests an underlying disorder related to dysregulated immune signaling pathways.

A limitation of this report is that TCRαβ^+^ double-negative T (DNT) cells were assessed only after sirolimus had been initiated. Because sirolimus may influence T-cell differentiation, the measured DNT frequency may not accurately reflect the pretreatment immunophenotype. Consequently, ALPS could not be formally excluded on the basis of pre-treatment DNT assessment alone.

Two years after treatment, despite marked clinical improvement, the patient continued to exhibit persistent immunologic abnormalities, particularly reduced CD4^+^ T cells and impaired T-cell differentiation. Analysis showed an almost complete loss of naive CD4^+^ and CD8^+^ T cells, accompanied by a marked expansion of CD4^+^ and CD8^+^ TEMRA populations, consistent with recent reports of skewed effector differentiation and depletion of naive T-cell compartments in APDS (13). This immunophenotypic profile reflects persistent PI3Kδ-driven T-cell dysregulation, in which aberrant activation of the PI3K–AKT–mTOR pathway promotes accelerated differentiation and reduces the naive T-cell pool (14).

These findings reflect the underlying APDS pathophysiology, in which hyperactivation of the PI3Kδ–AKT–mTOR pathway drives excessive T-cell activation and accelerated differentiation, leading to impaired immune renewal. In contrast, T-cell differentiation is generally preserved in classical HIGM, further supporting the diagnosis of APDS.

In this case, TCRαβ^+^ double-negative T (DNT) cells were assessed only after sirolimus had been initiated. As sirolimus may affect T-cell differentiation, the measured DNT frequency may not accurately represent the pretreatment immunophenotype. Therefore, ALPS could not be formally excluded based on pre-treatment DNT assessment alone. However, serial complete blood counts obtained during the year before sirolimus initiation consistently showed normal absolute lymphocyte counts (1.9–2.7 × 10^9^/L) without persistent lymphocytosis. Although this does not replace pre-treatment DNT assessment, the absence of persistent lymphocytosis is not consistent with the typical hematologic profile of ALPS. At the 2-year follow-up, TCRαβ^+^ double-negative T cells were not expanded. Together with the identification of a pathogenic PIK3CD variant, these findings favored a diagnosis of APDS over ALPS.

Regarding the immunophenotypic analysis, the most striking finding in our patient was the severe exhaustion of the naive T-cell pool (0.16% for CD8^+^ and 0% for CD4^+^), accompanied by a massive expansion of the TEMRA population (41.77% in CD8^+^ and 91.85% in CD4^+^). This specific cellular distribution is a hallmark of APDS, reflecting an accelerated state of T-cell senescence. According to the diagnostic framework recently proposed by Bozkurt, an increased percentage of CD8^+^CD45RA^+^CCR7^-^ (TEMRA) cells is a major laboratory indicator, present in approximately 50% of confirmed APDS cases (13). This accumulation of terminally differentiated cells results from the constitutive overactivation of the PI3Kδ pathway, which forces lymphocytes to bypass normal maturation and enter premature senescence. It is noteworthy that while sirolimus therapy successfully controlled the patient’s clinical symptoms—such as hepatosplenomegaly and recurrent infections—it did not fully restore the T-cell compartment. The persistence of this TEMRA-dominant profile suggests that the underlying immune dysregulation remains a long-term challenge, explaining why the patient continues to require immunoglobulin replacement therapy to maintain protective immunity.

Lymphoproliferation is a common manifestation in activated PI3Kδ syndrome (APDS) and may lead to suspicion of lymphoma. Cohort studies and case reports have described that APDS patients often manifest chronic lymphoproliferation, while malignant lymphoma has been recorded only in some cases (2, 3, 15). In our case, suspicion of Hodgkin lymphoma was raised because of prolonged lymphadenopathy with hepatosplenomegaly.

However, the clinical course, with lymph nodes appearing as early as infancy and persisting for many years without typical signs of malignant progression, together with the results of in-depth pathology consultation, helped exclude lymphoma at the time of biopsy. The lesion adjacent to the pancreatic head indentified on CT scan and abdominal ultrasound was most consistent with lymphoid tissue and resolved almost completely after 3 months of treatment.

A limitation of this report is that the lesion adjacent to the pancreatic head was not histologically confirmed. The diagnosis was based on imaging findings, multidisciplinary radiologic review, and its rapid resolution after sirolimus treatment.

This case emphasizes that in patients with early and prolonged lymphoproliferation on the background of immunodeficiency, APDS should be considered as an important differential diagnosis before concluding lymphoma. At the same time, lymph node biopsy and histopathologic evaluation by experienced experts play an important role in differentiating benign APDS-related lymphoproliferation from malignant lymphoma, helping avoid overtreatment.

Although our patient had detectable EBV viremia, chronic EBV infection is an important concern in APDS because persistent EBV replication has been associated with lymphoproliferation and an increased risk of lymphoma (3). Findings from the ESID-APDS Registry further supported the association between chronic EBV viremia and B-cell malignancy, emphasizing the importance of longitudinal EBV DNA monitoring in these patients (6). In line with the 2024 IUIS classification, APDS remains recognized as an inborn error of immunity with marked immune dysregulation and susceptibility to malignancy (1). Although sirolimus may help control lymphoproliferative manifestations, it does not completely eliminate the risk of malignant transformation (3, 16). Therefore, our patient continues to require close follow-up for progressive lymphadenopathy, worsening hepatosplenomegaly, hematologic abnormalities, and changes in EBV viral load (17).

The *PIK3CD* c.3061G>A, p.(Glu1021Lys) has been reported as the most common variant in large APDS cohort reports. The absence of the variant in both parents further supports its pathogenicity. In a large cohort of 53 APDS patients reported by Coulter and colleagues, the p.E1021K variant is the most common mutation in the *PIK3CD* gene. Patients carrying this variant had prominent immunologic characteristics including increased IgM (78%), decreased IgG (43%), and reduced CD4^+^ T-cell count (84%), together with high rates of bronchiectasis (60%) and lymphoproliferation (75%). These features are consistent with the immune abnormalities and clinical manifestations recorded in our patient.

Similar to patients with the same p.E1021K variant in the cohorts of Angulo and Lucas, our patient also had manifestations of recurrent respiratory infections, early onset, accompanied by chronic lung lesions and bronchiectasis (2, 15). These are prominent and classic clinical characteristics of APDS in most reports. In addition, the patient also had humoral immunodeficiency with reduced IgG and IgA recorded in most patients in Angulo’s cohort.

However, compared with patients in these cohorts, our patient more clearly expressed phenotypes similar to HIGM with persistently high IgM levels while IgG and IgA were severely reduced. In the studies of Angulo and Lucas, although increased IgM was recorded in some patients, it was not a prominent characteristic in all cases. These studies analyzed that this IgM increase was not due to an absolute defect of class-switch recombination, but reflected dysregulation of B-cell differentiation on the background of excessive activation of PI3Kδ signaling. This is supported by the observation that patients’ B cells still retain the ability to generate IgG under *in vitro* stimulation, along with increased transitional B cells and plasmablasts. Therefore, our case contributes to strengthening the view that APDS, especially the p.E1021K variant, may present an HIGM-like phenotype from the early stage and be easily confused with classical HIGM (2).

Although responses to sirolimus are variable across studies, our patient achieved sustained clinical improvement over a 2-year follow-up, with no adverse effects observed. However, follow-up immunophenotyping demonstrated persistent T-cell abnormalities, including naive T-cell depletion and TEMRA expansion, which may explain the incomplete immunologic response to mTOR inhibition and the continued dependence on immunoglobulin replacement. These findings suggest that sirolimus provides effective control of lymphoproliferation but acts as a disease-modifying rather than curative therapy in APDS.

The systematic analysis by Hanson and Bonnen, on 256 APDS patients, showed that the overall prognosis of APDS remains worse than the general population, with survival declining with age and lymphoma being the leading cause of death, followed by complications related to hematopoietic stem cell transplantation (8). Moreover, in the study by Whalen and colleagues of 37 APDS patients treated with leniolisib, 10/27 patients (37%) who had an indication for IgRT reduced or discontinued immunoglobulin replacement after leniolisib treatment (18). This is a promising direction, with the potential to significantly improve antibody deficiency in APDS patients. Therefore, we plan to continue sirolimus therapy, while reserving hematopoietic stem cell transplantation for severe or treatment-refractory disease. PI3Kδ-targeted therapy may also be considered if it becomes available.

## 4 Conclusion

APDS is a rare inborn error of immunity, with a risk of multi-organ involvement and easy confusion with HIGM. In children presenting with early-onset infections, elevated serum IgM, the presence of features atypical for classical HIGM, particularly chronic lymphadenopathy, chronic hepatosplenomegaly, or gastrointestinal involvement and reduced B-cell count, APDS should be actively considered and genetic testing for *PIK3CD* variants should be performed. Appropriate mechanism-based therapy may help control lymphoproliferation, prevent progressive organ damage, and improve clinical outcomes compared with conventional management for HIGM.

## Data Availability

The original contributions presented int he study are included in the article/supplementary material, further inquiries can be directed to the corresponding author/s.
